# GlnR Activation Induces Peroxide Resistance in Mycobacterial Biofilms

**DOI:** 10.3389/fmicb.2018.01428

**Published:** 2018-07-04

**Authors:** Yong Yang, Jacob P. Richards, Jennifer Gundrum, Anil K. Ojha

**Affiliations:** ^1^Division of Genetics, Wadsworth Center, New York State Department of Health, Albany, NY, United States; ^2^Department of Infectious Diseases and Microbiology, University of Pittsburgh, Pittsburgh, PA, United States; ^3^Department of Biomedical Sciences, University at Albany, Albany, NY, United States

**Keywords:** mycobacteria, biofilms, peroxide, starvation, GlnR

## Abstract

Mycobacteria spontaneously form surface-associated multicellular communities, called biofilms, which display resistance to a wide range of exogenous stresses. A causal relationship between biofilm formation and emergence of stress resistance is not known. Here, we report that activation of a nitrogen starvation response regulator, GlnR, during the development of *Mycobacterium smegmatis* biofilms leads to peroxide resistance. The resistance arises from induction of a GlnR-dependent peroxide resistance (*gpr*) gene cluster comprising of 8 ORFs (MSMEG_0565-0572). Expression of *gpr* increases the NADPH to NADP ratio, suggesting that a reduced cytosolic environment of nitrogen-starved cells in biofilms contributes to peroxide resistance. Increased NADPH levels from *gpr* activity likely support the activity of enzymes involved in nitrogen assimilation, as suggested by a higher threshold of nitrogen supplement required by a *gpr* mutant to form biofilms. Together, our study uniquely interlinks a nutrient sensing mechanism with emergence of stress resistance during mycobacterial biofilm development. The *gpr* gene cluster is conserved in several mycobacteria that can cause nosocomial infections, offering a possible explanation for their resistance to peroxide-based sterilization of medical equipment.

## Introduction

Under most detergent-free *in vitro* conditions, mycobacterial species grow as surface-associated, three-dimensionally organized multicellular communities, called biofilms, which develop through dedicated genetic programs (Ojha et al., 2005, 2008; Weiss and Stallings, 2013; Gupta et al., 2015; Chuang et al., 2016; Yang et al., 2017; Clary et al., 2018). Biofilm-like multicellular aggregates of non-tuberculous mycobacteria (NTMs) have also been reported from clinical and environmental specimens (Feazel et al., 2009; Bosio et al., 2012; Mullis and Falkinham, 2013; Fennelly et al., 2016). Biofilms of *Mycobacterium avium*, a prominent member of NTMs, have been implicated in pathogenesis (Rose and Bermudez, 2014), although biofilms of other pathogenic mycobacterial species including *M. tuberculosis* in the context of their host environments remain to be evaluated. Further clinical significance of mycobacterial biofilms is highlighted by at least two unique phenotypes, which are not associated with their single-cell planktonic counterparts. First, mycobacterial biofilms harbor a sizable subpopulation of bacilli that can survive extreme conditions including exposure to antibiotics and antiseptics (Falkinham, 2007; Ojha et al., 2008; Rose et al., 2015; Yang et al., 2017; Clary et al., 2018). Second, biofilm growth of some mycobacterial species, including the pathogenic species *M. canettii*, fosters horizontal gene transfer (Nguyen et al., 2010; Boritsch et al., 2016), which possibly accelerates the propagation of drug resistance mutations in these species. Although mycobacterial biofilms are increasingly being recognized as potential targets for effective anti-mycobacterial strategies, mechanisms underlying the emergence of stress tolerance in biofilms remain unknown.

Biofilm development in the model mycobacterial species, *M. smegmatis*, is a genetically programmed process that appears to occur in distinct stages, each demarcated by its specific genetic requirements (Ojha et al., 2005; Ojha and Hatfull, 2007; Yang et al., 2017). While the cell surface glycopeptidolipid (GPL) is necessary for optimum substratum attachment, a nucleoid-associated protein, Lsr2, is required for cell–cell aggregation (Yang et al., 2017). Moreover, gene expression analysis of an extragenic suppressor of an *lsr2* mutant revealed that cell–cell aggregation is a critical checkpoint in the developmental process (Yang et al., 2017). Expression levels of 83 genes are dependent on intercellular aggregation and aggregated growth (Yang et al., 2017), suggesting that the physicochemical interactions among cells induce transcriptional reprogramming for further maturation of architecture and physiological adaptation of resident cells.

A large number of 83 aggregation-dependent genes are under the control of GlnR, a conserved OmpR-like transcription factor that regulates nitrogen assimilation in response to its limited availability (Amon et al., 2008; Jenkins et al., 2013; Yang et al., 2017). GlnR-dependent upregulation of three ammonium transporters (Amt), glutamine/glutamate synthases (GlnA) and nitrite/nitrate reductases facilitate efficient assimilation of environmental nitrogen in a cell (Amon et al., 2008; Yang et al., 2017). In addition, GlnR also induces urecase, amidase, xanthin permeases, which likely maximize the total intracellular nitrogen pool (Jenkins et al., 2013). However, the fact that GlnR induces over 100 genes in mycobacteria opens up questions about its wider influence in mycobacterial growth and adaptation. Studies in other species support a global role of GlnR, extending beyond nitrogen assimilation. In *Saccharopolyspora erythraea,* GlnR controls the expression of carbohydrate ATP-binding cassette (ABC) transporters, thereby facilitating carbon uptake in response to nitrogen starvation (Liao et al., 2015). Similarly, GlnR also appears to control the expression of a key phosphate-sensing regulator, PhoP, in *S. erythraea,* implying a possible role of GlnR in phosphorous homeostasis (Yao and Ye, 2016). In addition to controlling nutrient balance in bacteria, GlnR also influences secondary metabolism in actinomycetes. In *Streptomyces coelicolor* and *Streptomyces avermitilis*, GlnR modulates the synthesis of antibiotics by directly regulating the transcription of pathway-specific genes (He et al., 2016; Urem et al., 2016). Lastly, GlnR also controls osmolyte levels in *S. coelicolor* (Shao et al., 2015), and pH homeostasis in *Streptococcus salivarius* (Huang and Chen, 2016).

Given a global role of GlnR in other species, we asked whether its activation during biofilm development in *M. smegmatis* has any significance beyond nitrogen assimilation. We report here that GlnR activation for nitrogen assimilation during biofilm growth also increases resistance to peroxide. The phenotype is caused by induction of a GlnR-dependent peroxide resistance (*gpr*) cluster of genes. The *gpr* cluster is comprised of 8 open reading frames (ORFs) – MSMEG_0565-0572– encoding genes of diverse functions. The upstream region of this uncharacterized operon has binding sites for both GlnR and SoxR, which is a MarR-family transcription factor that responds to oxidative stress to maintain redox homeostasis (Dietrich et al., 2008; Jenkins et al., 2013). However, *gpr* induction responds differently to GlnR and SoxR activities. While GlnR is a strong positive regulator of *gpr*, inducing it by ∼100-fold under limiting nitrogen, SoxR is a modest negative regulator causing twofold de-repression under peroxide stress. Emergence of peroxide resistance through a nutrient sensing mechanism provides a direct link between form and function of mycobacterial biofilms.

## Materials and Methods

### Bacterial Strains and Growth Conditions

All plasmids and strains used in this study are listed in Supplementary Tables S1 and S2, respectively. Unless indicated, *M. smegmatis*, mc^2^155 (wild-type), was maintained at 37°C in 7H9ADC (Becton Dickinson) with 0.05% (v/v) Tween-80 for planktonic cultures. 7H10ADC agar (Becton Dickinson) was used for plate cultures. When necessary, hygromycin, kanamycin, and zeocin were added at 150, 20, or 25 μg/mL, respectively, to culture recombinant strains. *Escherichia coli* (DH5α) was grown at 37°C in LB broth or LB agar. Pellicle biofilms of *M. smegmatis* strains were grown as described earlier (Yang et al., 2017). Briefly, 10 μL of saturated planktonic cultures were inoculated into 10 mL of detergent-free Sauton’s medium or modified M63 medium in either 60 mm polystyrene dishes or 12-well polystyrene plates, and incubated stationary at 30°C untill indicated time. The N_0_ version of Sauton’s medium was prepared by omitting asparagine, and by replacing the ferric ammonium citrate with ferric citrate. The N_1/2_ version was prepared by reducing the initial concentrations of the above mentioned nitrogen sources by half.

### Construction of Mutants and Plasmids

Mutations in *M. smegmatis* mc^2^155 were constructed using recombineering as described previously (Yang et al., 2017). Briefly, allelic exchange substrates for a given target gene were generated by SOEing-PCR on either side of a *loxP* flanked zeocin-resistant cassette using the respective primers listed in Supplementary Table S3. The purified PCR-products were electroporated into an electrocompetent recombineering strain, mc^2^155-pJV53-SacB, and plated on 7H10ADC with 25 μg/mL zeocin. Mutant genotypes of *zeo^r^* colonies were confirmed by PCR. The recombineering plasmid, pJV53-SacB, was rescued from mutants by plating them on 7H10ADC with 15% sucrose, and screening sucrose resistant colonies for kanamycin sensitivity. The *zeo^r^* marker was removed by excision using a Cre recombinase expressed from pCre-SacB, which was electroporated into the rescued mutant cells and transformants were screened for loss of *zeo^r^*. The *zeo^s^* colonies were screened on 7H10ADC with 15% sucrose to obtain clones without the pCre-SacB plasmid. The rescued unmarked mutants were complemented as indicated.

### RNA-seq

*Mycobacterium smegmatis* mc^2^155 and Δ*glnR* were grown in detergent-free Sauton’s medium to form matured pellicle biofilms. Total RNA was extracted using a Qiagen RNeasy kit and contaminating DNA was removed with the turbo DNA-free kit (Thermo Fisher Scientific). For each sample, 5 μg of total RNA was processed for rRNA removal using the Ribo Zero kit (Illumina). Strand-specific DNA libraries were then prepared with 100 ng of mRNA using the Scriptseq Complete Kit- Bacteria (Illumina). Libraries were sequenced on the NextSeq500 platform (Illumina) and analyzed by Rockhopper (McClure et al., 2013) at default settings using the reference genome of *M. smegmatis* mc^2^ 155 (NC_008596).

### RT-qPCR

All oligonucleotides used for RT-qPCR are listed in Supplementary Table S3. DNA-free RNA for RT-qPCR was extracted as described for RNA-seq. For each sample, 200 ng of RNA was used for reverse transcription using the Maxima First Strand cDNA Synthesis Kit (Thermo Fisher Scientific). RT-qPCR was performed on an Applied Biosystems 7000 fast RT-qPCR System (Applied Biosystems) with SYBR Green Master Mix following the manufacturer’s instructions. Relative expression of target gene is calculated either as 2^-ΔCt(gene-SigA)^or2^-ΔCt(targetgene1-SigA)-ΔCt(targetgene2-SigA)^, in which SigA transcript was the endogenous control.

### Antibiotics and Peroxide Sensitivity Assays

Exponential phase culture (OD_600_ = 0.3) of each strain grown in Sauton’s medium with 0.05% (v/v) Tween80 was harvested and washed once with phosphate buffered saline with 0.05% Tween-80 (PBST). Approximately 2 × 10^7^ CFU/mL of each strain was resuspended in nitrogen-free (N_0_) Sauton’s medium with 0.05% (v/v) Tween80 at 37°C for 3 h. 400 μg/mL rifampicin, 1 μg/mL streptomycin or 20 mM H_2_O_2_ were added and incubated at 37°C for the indicated period; unexposed cultures were used as control. At the indicated time point, the exposed and unexposed cultures were diluted and plated on 7H10ADC for bacterial viability.

### Measurement of Intracellular NADPH/NADP Ratio

Average intracellular NADPH/NADP ratios of planktonic culture and biofilms were measured according to previous publication with minor modifications (Vilcheze et al., 2005). Briefly, exponential phase planktonic and biofilm cultures from normal and N_0_ Sauton’s medium were harvested and washed once with PBS and then resuspended in PBS. Large aggregates from biofilms were broken up by 8–10 repeated passaging through 18-G needles. 1.5 mL single cell suspensions at density of 10^8^–10^9^ CFU/mL of each strain were pelleted and resuspended in 0.75 mL 0.2 M HCl (for NADP extraction) or 0.75 mL of 0.2 M NaOH (for NADPH extraction). After 10 min at 55°C, the suspensions were cooled to 0°C and neutralized by adding either 0.75 mL of 0.1 M NaOH for NADP extraction or 0.75 mL of 0.1 M HCl for NADPH extraction, while vortexing at high speed. After incubation for 10 min on ice, the suspensions were centrifuged at 3000 rpm for 10 min at 4°C. The supernatants were filtered and transferred to a new tube and used immediately. The concentrations of NADP and NADPH in the suspensions were determined by spectrophotometric measurement of the rate of 3-[4,5-dimethylthiazol-2-yl]-2,5-diphenyltetrazolium bromide (Sigma # M2128) reduction by the glucose-6-phosphate dehydrogenase (Sigma # G6378) in the presence of phenazine ethosulfate (Sigma # P4544) at 570 nm. The rate of 3-[4,5-dimethylthiazol-2-yl]-2,5-diphenyltetrazolium bromide reduction is proportional to the concentration of the nucleotides. Purified NADP (Sigma # 10128031001) and NADPH (Sigma # 10107824001) were used for standard curves, which were used for determination of the nucleotides in each sample. Serial dilutions of samples were tested to ensure the values were in the linear range of the NADP/NADPH standard curve.

### Peroxide Sensitivity Assay for Microfluidic Biofilms

Biofilms as microcolonies were grown in a CellASIC ONIX (Cat # EV262) microfluidic platform, using CellASIC microfluidic plates (M04S-03) with headspace of 150 μm. Approximately 10^6^ CFU/mL of bacteria in 10 μL media were perfused at a pressure of 1.7 kPa (0.25 psi) into each culture chamber of the plate for 6 s, followed by no perfusion for 30 min. This allowed optimum attachment of single cells to the culture chamber surface at a density that then grew into separate colony biofilms. For microfluidic culturing, detergent-free Sauton’s media was perfused across each culture chamber at the manufacturer’s recommendation of a dual pressure of 3.4 kPa (0.5 psi) at 37°C for 4 days to provide adequate nourishment and minimal stress to biofilm-like colonies. Incubation of Δ*glnR* was extended by an additional day to allow colony biofilms to grow to the same size as wild type. Colony biofilms were exposed to 20 mM H_2_O_2_ in Sauton’s media via microfluidic perfusion for 3 h followed by overnight perfusion of Sauton’s media with 1 μg/mL Calcein AM to stain survivors. Images of colony biofilms were collected by confocal laser scanning microscopy (CLSM) at 20× magnification under green channel (excitation 488 nm). Corresponding DIC image of each colony was also captured for overlaying the fluorescence signal. The images were analyzed by ImageJ. To compare the numbers of surviving cells among strains after H_2_O_2_ exposure, the number of green CalceinAM-stained cells was calculated from the maximum intensity projection of the z-stacks of each biofilm-like colony, normalized to colony surface area. For each strain, at least three biofilm-like colonies over two fields of view were analyzed.

## Results

### GlnR and Biofilm Formation in *M. smegmatis*

**FIGURE 1:**
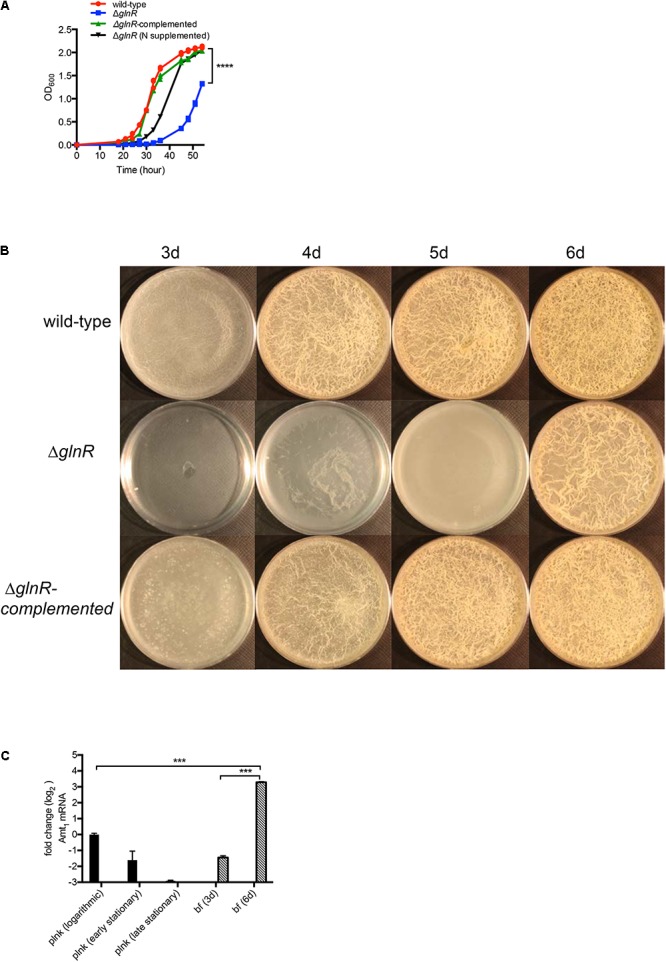
GlnR-dependent growth of *M. smegmatis* in planktonic and biofilm cultures in Sauton’s medium. **(A)** Planktonic growth of wild-type, Δ*glnR* and Δ*glnR*-complemented strains in Sauton’s medium with 0.05% (v/v) Tween80. Growth of Δ*glnR* is also rescued by supplementation of Sauton’s medium with 0.5% (w/v) casamino acid and 0.2% (w/v) ammonium sulfate. **(B)** A top-down view of pellicle biofilms of wild-type, Δ*glnR* and Δ*glnR* complemented strains in detergent-free Sauton’s medium at the indicated time point. **(C)** Expression of a GlnR-dependent ammonium transporter (Amt_1_) in planktonic (plnk) and biofilm (bf) cultures of wild-type *M. smegmatis* at indicated stages of growth. Logarithmic, early- and late-stationary phases of planktonic cultures correspond to OD 0.3, 1.5 and 2.5, respectively. Stages of biofilms at which cells were harvested are indicated as days after incubation. Expression was determined by real-time PCR and normalized with the SigA transcripts. Data represent mean of two independent experiments. ^∗∗∗^ and ^∗∗∗∗^ denote *p* < 0.001 and 0.0001, respectively (*t*-test).

In our earlier study we identified 61 genes induced during maturation of *M. smegmatis* biofilms to be GlnR-dependent (Jenkins et al., 2013; Yang et al., 2017). We therefore tested the effect of *glnR* mutation on development of *M. smegmatis* biofilms. Deletion of *glnR* produced no apparent phenotype in modified M63 medium, which was used in our earlier study (Yang et al., 2017) (Supplementary Figure S1). However, the mutation caused delayed planktonic growth and retarded biofilm development in Sauton’s medium (**Figures 1A,B**), which has poorer nitrogen source relative to the modified M63 medium. Lack of adequate nitrogen source in Sauton’s medium appeared to be the primary cause for Δ*glnR* phenotype, because the mutant growth could be substantially rescued by addition of casamino acid as supplemental nitrogen source (**Figure 1A**). Moreover, similar to the observation in modified M63 medium (Yang et al., 2017), late-stage (6-day) biofilms of wild-type (mc^2^155) *M. smegmatis* in Sauton’s medium also exhibited > 50-fold induction of a GlnR-dependent ammonium transporter (MSMEG_2425; Amt_1_) (**Figure 1C**). The induction was not observed in planktonic culture of the strain (**Figure 1C**), indicating that nitrogen availability in the medium is sufficiently high to prevent peak level of GlnR activation in planktonic cells, but not high enough to do so in biofilms. Based on these findings, and to maintain consistent growth conditions used in previous studies of GlnR mutant of *M. smegmatis* (Jenkins et al., 2013), we chose to use Sauton’s medium for this study.

Growth retardation of Δ*glnR* in planktonic cultures (**Figure 1A**) suggests that a basal activity of the regulator is necessary for optimum uptake of nitrogen sources. Delayed but matured biofilm development by Δ*glnR* raised the possibility of either an alternative mechanism of induction of GlnR-dependent genes, or existence of alternative pathways for nitrogen assimilation. To investigate these possibilities we compared the transcriptomes of 6-day biofilms of wild-type and Δ*glnR* strains. Expression of GlnR-dependent genes was significantly retarded in biofilms of Δ*glnR* mutant, compared to the wild-type (Supplementary Table S4), suggesting that secondary mechanisms of nitrogen assimilation are triggered in the mutant. This was further substantiated by upregulation in Δ*glnR* mutant biofilms of acetamidase (*amiE* or MSMEG_5335) and D-amino acid dehydrogenase (Supplementary Table S4).

### GlnR Activation and Peroxide Resistance

**FIGURE 2:**
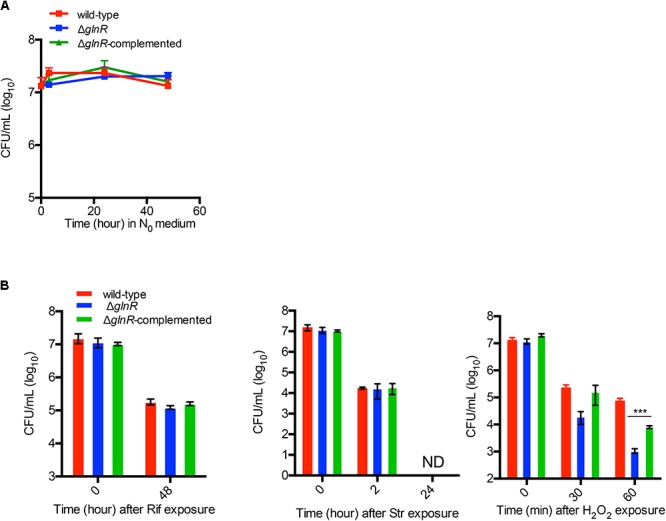
GlnR-dependent resistance of *M. smegmatis* to hydrogen peroxide under nitrogen-limiting condition. **(A)** Survival of wild-type, Δ*glnR* and Δ*glnR*-complemented strains in nitrogen-free Sauton’s (N_0_) medium for up to 48 h. Exponentially growing cells (OD 0.3) cultured in Sauton’s medium with 0.05% (v/v) Tween80 were washed and resuspended in N_0_ medium for indicated time points prior to plating the dilutions on 7H10ADC plate. **(B)** Effect of streptomycin (Str; 1 μg/mL), rifampicin (Rif; 400 μg/mL), and hydrogen peroxide (H_2_O_2_; 20 mM) on survival of wild-type, Δ*glnR* and Δ*glnR* -complemented strains in N_0_ medium. Cells were incubated in N_0_ medium for 6 h prior to exposure to each condition for the indicated period of time. Data are representative of mean of three biologically independent experiments. ^∗∗∗^ indicates *p* (*t*-test) < 0.001.

A global effect of GlnR on gene expression patterns (Jenkins et al., 2013; Jessberger et al., 2013; Yang et al., 2017) raised a possibility that its activation during adaptation of *M. smegmatis* to low nitrogen may also impact other functions of cells. We investigated the effect of GlnR on persistence under stress exposure by comparing the sensitivity of nitrogen-starved cells of wild-type, Δ*glnR* and Δ*glnR* complemented strains to two commonly used anti-TB antibiotics: rifampicin (Rif) and streptomycin (Str) at 10X MIC. We also included hydrogen peroxide – a routinely used sterilizing agent for control of biofilm-related contaminants of surgical equipment in nosocomial settings (Falagas et al., 2011). We excluded isoniazid (INH) due to its selective activity on growing cells. We chose to test GlnR-activated planktonic cells by exposing them to N_0_-Sauton’s medium. GlnR was activated within 3 h of exposure to N_0_-Sauton’s medium (Supplementary Figure S2). Because viability of Δ*glnR* remains unaltered during the first 48 h of exposure to the N_0_-Sauton’s medium (**Figure 2A**), the exposure periods to the stressors were kept within this time limit. All three strains appeared equally sensitive to high concentrations of Rif and Str (**Figure 2B**). Interestingly, Δ*glnR* mutant showed significantly greater sensitivity to peroxide exposure in a 60-min period (**Figure 2B**), and the phenotype was substantially reversed in the complemented strain (**Figure 2B**).

### Peroxide Resistance Arises From GlnR-Dependent Induction of *gpr*

**FIGURE 3:**
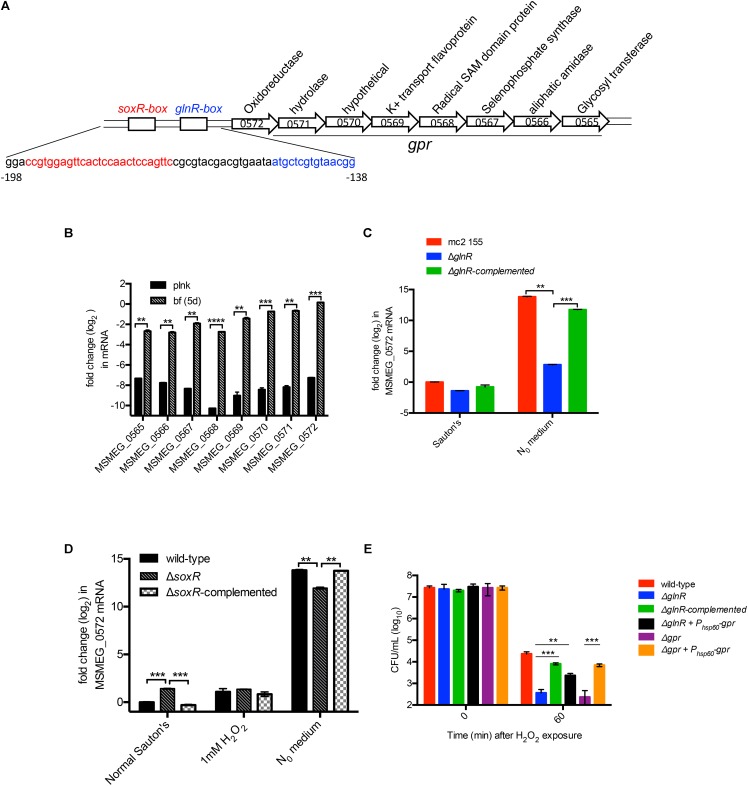
GlnR-dependent peroxide resistance in *M. smegmatis* results from activation of a cluster comprising of eight genes (MSMEG_0565 to 0572), called *gpr*. **(A)** Schematic representation of *gpr* and the upstream GlnR and SoxR-binding regions, called *GlnR*-box and *SoxR*-box, respectively. Nucleotide sequence of the two regions are indicated below in their corresponding color codes. **(B)** Expression of each of the eight genes in *gpr* cluster in logarithmic phase (OD 0.3) planktonic culture and in 6-day biofilms, both cultured in Sauton’s medium. Transcripts were normalized with SigA transcripts. **(C)** GlnR-dependent induced expression of *gpr* cluster (represented by MSMEG_0572) in N_0_ medium. Indicated strains were cultured in normal Sauton’s medium untill OD 0.3 and transferred to N_0_ medium for 3 h prior to mRNA analysis by real-time PCR. Cells collected from Sauton’s medium before exposure to N_0_ medium were used as reference. Fold-change relative to transcript level in wild-type cells in Sauton’s medium was calculated. **(D)** Expression of *gpr* (represented by MSMEG_0572) is unresponsive to SoxR and peroxide in Sauton’s medium. Indicated strains were cultured and processed as described for **(C)**, except that an additional set exposed to a sub-lethal concentration (1 mM) of H_2_O_2_ for 60 min was also included. **(E)** Expression of *gpr* is sufficient to restore peroxide resistance in Δ*glnR* mutant. Viability of the indicated strains before (0 min) and after 30 min of exposure to 20 mM H_2_O_2_. Data in **(B–E)** represent mean of three biologically independent experiments. ^∗∗^ and ^∗∗∗^ indicates *p* (*t*-test) < 0.01 and <0.001, respectively.

To determine the basis of GlnR-dependent peroxide resistance we analyzed the nucleotide sequence of GlnR-dependent genes. Upstream region of one of the GlnR-dependent operons comprising of 8 ORFs (MSMEG_0565 to MSMEG_0572) contained binding sites for both GlnR and SoxR (Dietrich et al., 2008; Jenkins et al., 2013) (**Figure 3A**). Since SoxR plays important role in redox homeostasis in many bacterial species (Storz and Imlay, 1999), we speculated that this locus could be under dual regulation of SoxR and GlnR, and that its activation by either of the two regulators possibly confers peroxide resistance. Biofilm-specific expression of all 8 ORFs in Sauton’s medium was verified by RT-qPCR (**Figure 3B**). We next tested the role of SoxR and GlnR in activation of the operon using MSMEG_0572 as a representative member. As expected from earlier studies (Jenkins et al., 2013; Jessberger et al., 2013; Yang et al., 2017), expression of the operon in wild-type cells was highly (>500 fold) induced upon 3-h exposure to N_0_ Sauton’s medium in a GlnR-dependent manner (**Figure 3C**). However, SoxR activity appears to have very modest (∼2-fold) negative effect on the induction of the operon (**Figure 3D**). Interestingly, exposure to peroxide did not induce the operon (**Figure 3D**). Together, these expression profiles indicate that regulation of the operon exclusively depends on GlnR under the tested conditions.

We next asked if expression of MSMEG_0565-72 operon is necessary and sufficient to exhibit GlnR-dependent peroxide resistance. A deletion mutant of the operon exhibited similar level of peroxide sensitivity as Δ*glnR* under N_0_-Sauton’s medium (**Figure 3E**), and the phenotype was rescued by plasmid-borne expression of the operon by a constitutive (P_hsp60_) promoter. Importantly, constitutive expression of the operon by P_hsp60_ promoter was also able to substantially rescue peroxide sensitivity of Δ*glnR,* indicating that peroxide resistance in *M. smegmatis* is primarily contributed by GlnR-dependent activation of MSMEG_0565-72 operon. We therefore call this operon as GlnR-dependent peroxide resistance or *gpr*.

**FIGURE 4:**
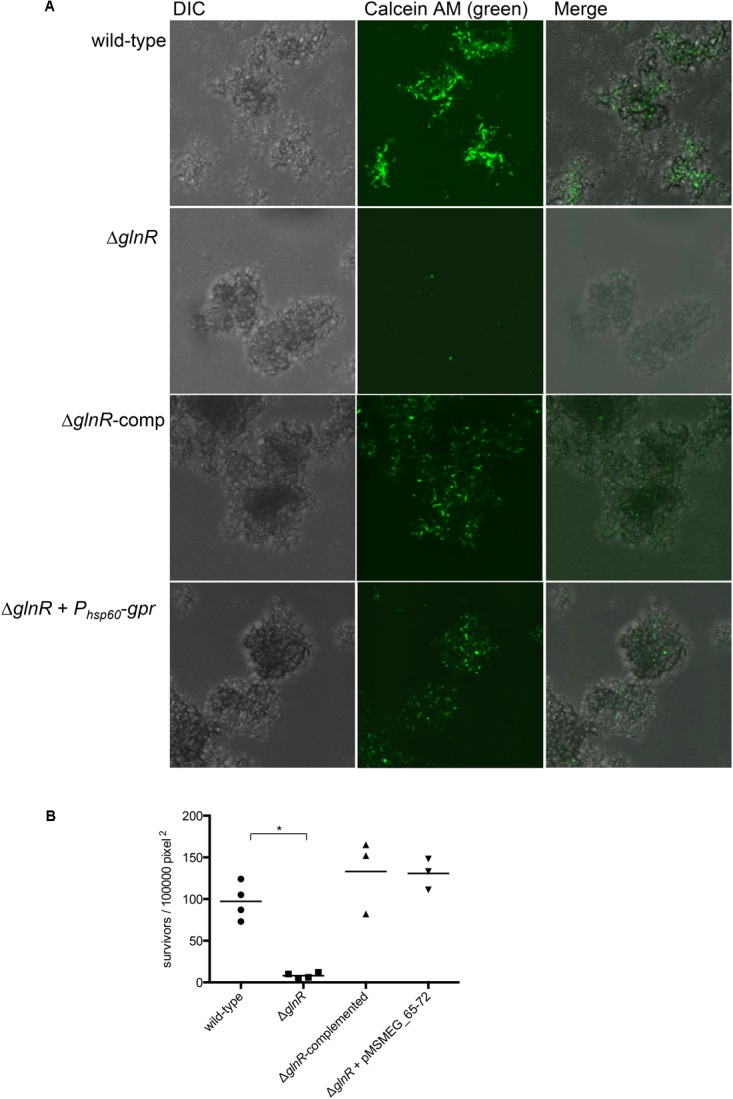
GlnR-dependent expression of *gpr* induces peroxide resistance in *M. smegmatis* biofilms. **(A)** Visualization of peroxide resistant survivors (green) in 4-day biofilms of the indicated strains. Biofilms, cultured in CellASIC Onix microfluidic system perfused with Sauton’s medium, were exposed with the medium containing 20 mM H_2_O_2_ for 3 h prior to staining with Calcein AM (1 μg/mL). Distributions of live cells (green) in colony biofilms were determined from images acquired by confocal microscopy. The micrographs represent maximum intensity projection of green signal across *z*-stacks analyzed by ImageJ. **(B)** A summary plot of frequency of green cells in four independent biofilms of wild-type and mutants, and three for the complemented strains. Data represent mean from 3 to 4 independent biofilms of each strain formed in a microfluidic chamber. ^∗^denotes *p* (Mann–Whitney) < 0.05.

To obtain a direct evidence for roles of *glnR* and *gpr* in peroxide resistance of *M. smegmatis* biofilms, we employed a microfluidic-based growth model to visualize surviving cells in peroxide exposed biofilms by confocal microscopy. To calibrate the growth model we first determined the timing of activation of GlnR by using a reporter strain of *M. smegmatis*, which harbored constitutively expressing mCherry and Dendra-2 fused to the promoter of Amt_1_ (MSMEG_2425). Expression of Dendra-2 in biofilms could be visualized after 4 days of growth in Sauton’s medium (Supplementary Figure S3). Subsequent incubation led to bacterial growth in the flow channels, leading to increased backflow pressure. We therefore used 4-day stage of wild type biofilms for our analysis, although biofilms of Δ*glnR* were cultured for an additional day to allow them to achieve similar size as wild-type. Following peroxide exposure, live cells in biofilms were probed by calcein AM, which remains non-fluorescent until its passive diffusion to the cytosol and hydrolysis by intracellular hydrolases produces fluorescent calcein (Rego et al., 2017). Compared to wild-type biofilms, the number of viable cells in peroxide exposed Δ*glnR* biofilms was significantly reduced (**Figures 4A,B**). The mutant phenotype could be complemented by plasmid-borne expression of either *glnR* from its native promoter or a constitutive expression of *gpr* from the hsp60 promoter. We thus conclude that induced expression of *gpr* upon activation of GlnR during maturation of *M. smegmatis* biofilms directly contributes to their peroxide resistance.

### Physiological Role of *gpr* in Biofilm Development

Nitrogen assimilation in majority of bacterial species occurs at the expense of the redox currency, NADPH (van Heeswijk et al., 2013), which serves as a co-factor for several enzymes, including glutamate synthase, involved in synthesis of ammonia and amino acids. Two subunits of NADPH-dependent glutamate synthase, encoded by MSMEG_3225 and MSMEG_3226, are induced in biofilms by ∼30-fold (Yang et al., 2017), and their induction is GlnR-dependent (Supplementary Table S4). This suggests a greater demand of NADPH for nitrogen-starved cells in biofilms. This is consistent with ∼5-fold increase NADPH/NADP ratio after 3 h of incubation of wild-type cells in N_0_-Sauton’s medium, relative to normal Sauton’s medium (Supplementary Figure S4). The increase in the ratio appears to be contributed by a modest decrease (<2-fold) in NADPH, relative to NADP (∼7-fold), suggesting that NADPH is likely regenerated from existing NADP by reductases induced in nitrogen-starved cells. Regeneration of NADPH, as opposed to new synthesis, is preferred perhaps due to lack of nutrients in N_0_-Sauton’s medium. We therefore hypothesized that induction of the putative reductases encoded by genes in *gpr* cluster likely regenerate NADPH pool to meet the metabolic demand of nitrogen-starved cells. Nitrogen-starved Δ*gpr* mutant indeed produced a lower NADPH/NADP ratio than wild-type and complemented cells (Supplementary Figure S4). The decreased ratio in the mutant was due to reciprocal change in NADP and NADPH levels, consistent with the idea that existing NADP are reduced to regenerate NADPH (Supplementary Figure S4).

**FIGURE 5:**
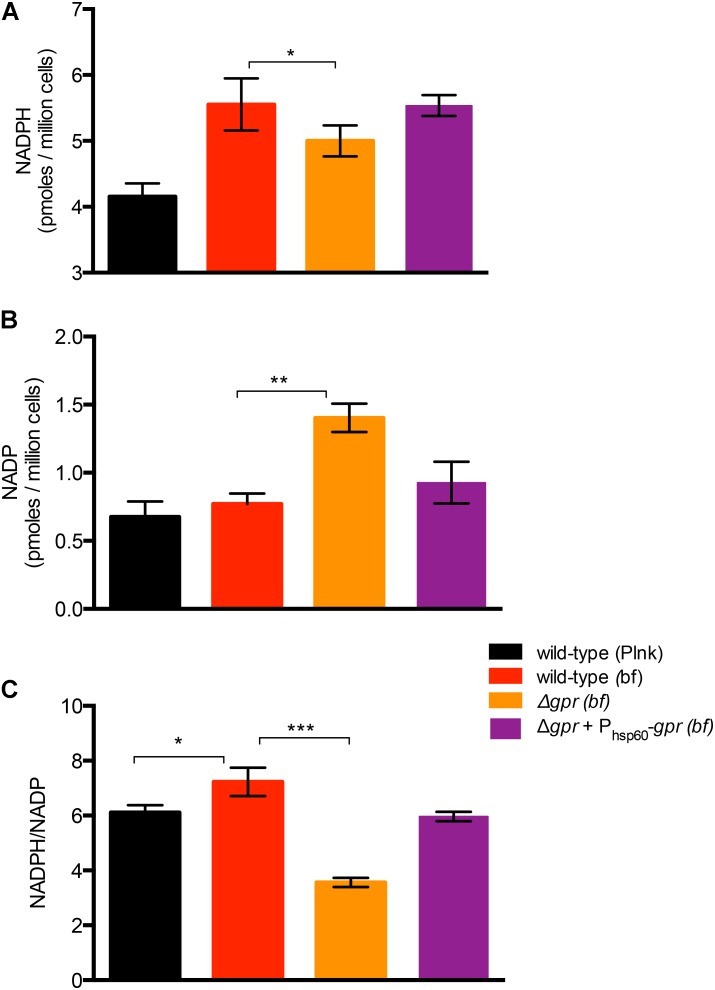
Role of *gpr* in maintenance of redox homeostasis of *M. smegmatis.* **(A,B)** Levels of oxidized (NADP) form of nicotinamide adenine dinucleotide phosphate in biofilms of wild-type, Δ*gpr* and the complemented strains cultured in normal Sauton’s medium. **(C)** Ratio of NADPH to NADP calculated from **(A,B)**. Data represent mean of three biologically independent experiments ^∗^, ^∗∗^, and ^∗∗∗^ indicate *p* (*t*-test) < 0.05, 0.01, and 0.001, respectively.

To test *gpr*-dependent redox homeostasis during biofilm development, we first compared the NADPH and NADP levels between planktonic and wild-type cells. Interestingly, the average NADPH/NADP ratio in biofilm cells of wild-type increased by ∼18%, relative to its planktonic counterpart, indicating that biofilm cells have a more reduced cytosolic environment (**Figure 5C**). The increase in the ratio resulted from a disproportionate increase in NADPH (∼34%), compared to NADP, which was maintained at a steady level (**Figures 5A,B**). This suggests increase in both new synthesis of NADP and its reduction. This was in contrast to the scenario observed in planktonic cells in N_0_-Sauton’s medium (Supplementary Figure S4). Expectedly, the average NADPH/NADP ratio in biofilms of Δ*gpr* mutant declined by nearly 50%, relative to wild-type, and the phenotype was substantially restored in the complemented strain (**Figures 5A–C**). Decline in the ratio in the mutant was due to accumulation of NADP, with concomitant decrease in NADPH (**Figures 5A–C**). Together, we infer that GlnR-dependent induction of *gpr* in a subpopulation of biofilm cells that experience nitrogen starvation is critical for maintenance of higher NADPH pool to meet their metabolic demand.

**FIGURE 6:**
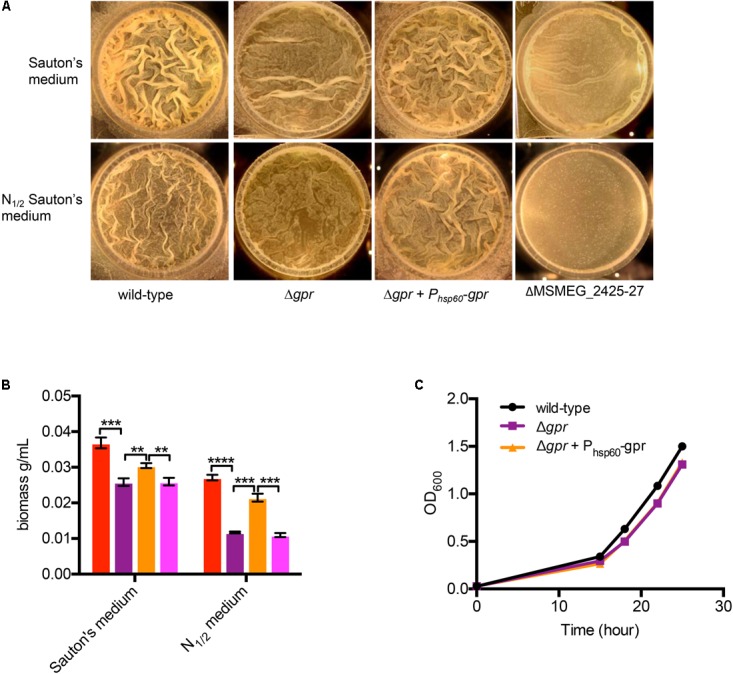
Role of *gpr* in biofilm formation of *M. smegmatis.* **(A)** A top-down view of pellicle biofilms of the indicated strains after days of growth in detergent-free Sauton’s medium and modified Sauton’s medium with half the normal nitrogen source (N_1/2_). In contrast to the wild type, which formed thick-textured biofilms in both medium, Δ*amt_1_* and Δ*gpr* mutants formed untextured biofilms in N_1/2_ Sauton’s medium, and the phenotype was partially rescued by nitrogen restoration in normal Sauton’s medium. **(B)** Biomass of the pellicles described in **(A)** from three independent experiments. The order of columns in the plot corresponds to the order in which the strains are indicated in **(A)**. Data represent mean of three biologically independent experiments. ^∗∗^, ^∗∗∗^, and ^∗∗∗∗^ denote *p* < 0.01, 0.001, and 0.0001, respectively (*t*-test). **(C)** Planktonic growth of the indicated strains in N_1/2_ Sauton’s medium.

A corollary to *gpr*-dependent redox homeostasis is that *Δgpr* mutant has impaired ability to assimilate nitrogen that impacts its growth in biofilms. Relative to wild-type, biofilm growth of *Δgpr* cells in normal Sauton’s medium was moderately retarded, but the effect was more pronounced in nitrogen-depleted (N_1/2_) Sauton’s medium, which has been shown to increase GlnR-dependency of *M. smegmatis* in biofilms (Yang et al., 2017) (**Figures 6A,B**). A mutant lacking a GlnR-dependent operon (MSMEG_2425-27), which was previously shown to display nitrogen-responsive biofilm defect (Yang et al., 2017), served as a reference in our analysis of Δ*gpr* phenotype (**Figures 6A,B**). Phenotype of Δ*gpr* could be complemented by plasmid-borne expression of *gpr* (**Figures 6A,B**). The defect was specific to biofilm culture, as no difference in planktonic growth of Δ*gpr* mutant was observed (**Figure 6C**).

Our preliminary attempts to identify specific gene(s) of *gpr* that could complement Δ*gpr* phenotype were unsuccessful (data not shown), suggesting that interaction between multiple genes of the cluster give rise to its function.

## Discussion

Mycobacteria express and utilize dedicated genes to build stress resistant biofilms (Richards and Ojha, 2014), raising a possibility that genetic programs involved in adaptation of resident cells within the architecture, and those in stress resistance overlap with each other. In this study, we provide evidence supporting this hypothesis by demonstrating a causal relationship between nitrogen-starvation response exhibited by biofilm cells and emergence of peroxide resistance in these cells. Peroxide resistance is likely a result of recalibration of NADPH/NADP ratio, skewed toward a more reduced cytosolic environment, to meet the increasing demand of NADPH for nitrogen assimilation in starved cells. These cells likely reside in interiors of biofilms, as suggested by localization of GlnR-activated cells in these regions of biofilms (Supplementary Figure S3). It is noteworthy that *E. coli* also acquire greater resistance to peroxide upon exposure to nitrogen starvation (Jenkins et al., 1988), although underlying mechanism remains unknown in this species.

The contribution of *gpr* cluster in maintenance of NADPH pool suggests specific function of encoded reductases in the process, although identity of these enzymes and their mechanisms remain open to further investigation. MSMEG_0572 appears to represent DsrE-family reductases, which are conserved in a wide range of environmental species of bacteria. Moreover, similar to MSMEG_0572, its orthologs in these species exhibit a syntenic arrangement with respect to the remaining seven genes of *gpr* cluster, suggesting that the entire cluster has migrated across genomes during evolution. This also raises the possibility that the cluster could function as a unit, rendering a possible explanation to our inability in identifying individual genes responsible for the phenotype associated with Δ*gpr* strain. Interestingly, one of the genes in the cluster, MSMEG_0567, is homologous to selenophosphate synthetase, which is directly involved in the synthesis of selenocysteine (Sec) (Turanov et al., 2011). Sec in prokaryotes is incorporated in polypeptide by a set of specialized accessory factors, which facilitate Sec-tRNA to decode the UGA codon (Krol, 2002). The UGA codon in mRNA decoded as Sec must have a downstream Sec insertion sequence (SECIS), which forms a unique stem-loop structure recognized by the accessory factors that recruit Sec-tRNA during translation (Krol, 2002). Bioinformatics search using bSECIS program (Zhang and Gladyshev, 2005) of all *gpr* ORFs identified a SECIS element comprising of 39 bp downstream of UGA codon of MSMEG_0571, suggesting that the codon in this ORF is decoded as Sec, thereby giving rise to a selenoprotein. The resulting selenoprotein is extended by 518 amino acids (aa) to terminate at the originally annotated stop codon (UAG) of MSMEG_0569 (Supplementary Figure S5). BLAST search of thus encoded 818aa long selenoprotein encompassing MSMEG_0567 to MSMEG_0569 reveals a conserved nitrilase-like domain in the N-terminal region and a flavin-dependent oxidoreductase domain in the C-terminal region (Supplementary Figure S5). This domain architecture is consistent with the fact that majority of the selenopoteins known so far are oxidoreductase (Hatfield et al., 2014), supporting the function of *gpr* cluster in redox homeostasis. The *gpr* cluster also includes a putative aliphatic amidase (MSMEG_0566), which could directly contribute to nitrogen assimilation.

**FIGURE 7:**
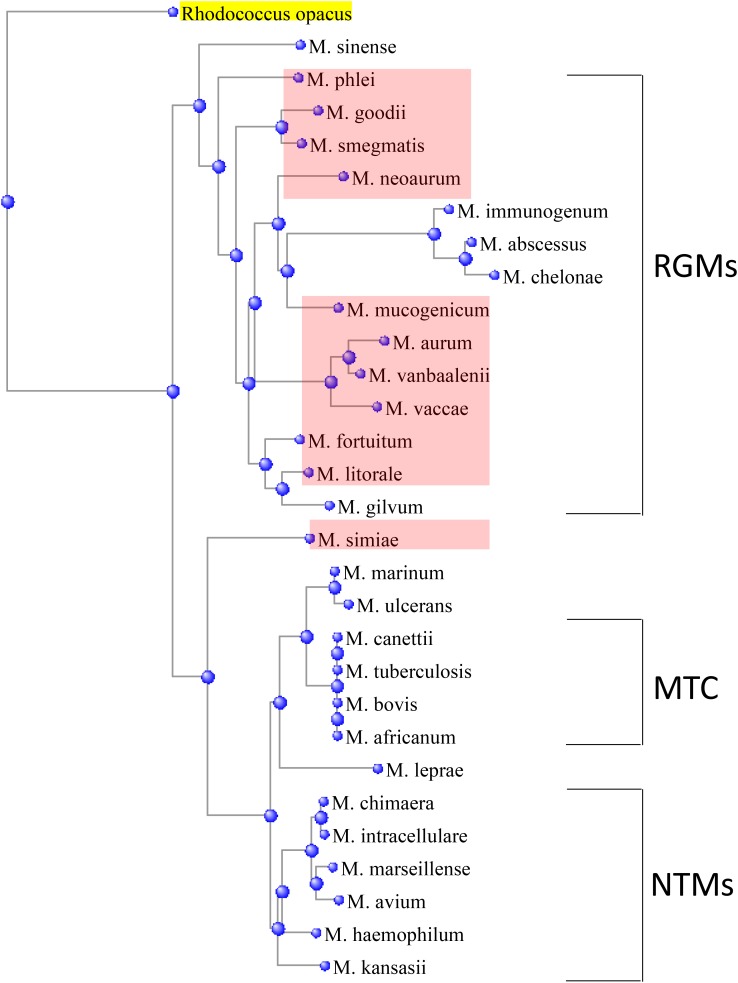
Presence of *gpr* locus in a subset (shaded pink) of rapidly growing mycobacteria (RGMs), which are depicted in relationship to the other RGMs, slow-growing non-tuberculous mycobacteria (NTMs), and *Mycobacterium tuberculosis* complex (MTC), based on their similarities in 16S rRNA sequences. A member of NTM, *Mycobacterium simiae,* is an outlier. The phylogenetic tree was constructed by distance method using *Rhodococcus opacus* as an outgroup.

A homology search for *gpr* cluster in 29 mycobacterial species reveals its presence in only a few members, which are evolutionarily linked based on 16S rRNA phylogeny (**Figure 7**). Most of these species are classified as rapidly growing mycobacteria (RGM) (Runyon, 1959). Interestingly, *gpr* is absent in a clinically important RGM, *M. abscessus*, suggesting that its transfer to RGM from a common ancestor is a relatively recent evolutionary event. Moreover, evidence of horizontal gene transfer is supported by the presence of *gpr* in the only slow-growing non-tuberculosis mycobacteria, *M. simiae* (**Figure 7**). Presence of *gpr* in *M. mucogenicum*, *M. goodii,* and *M. simiae,* which are emerging pathogens in nosocomial infections (van Ingen et al., 2008; Adekambi, 2009; Parikh and Grant, 2017; Salas and Klein, 2017), raises clinical significance of our findings and possibly offers insight into resistance of these species to peroxide-mediated sterilization of surgical and medical equipment.

Lack of *gpr* in a majority of mycobacteria suggests a different mechanism of GlnR-dependent nitrogen assimilation in these species, consistent with the differences between GlnR activities in *M. smegmatis* and *M. tuberculosis* as described previously (Williams et al., 2015). Further understanding of molecular underpinnings of these differences is likely to identify a role of GlnR in biofilm development and associated stress tolerance in these species.

## Author Contributions

AO and YY conceived this study. YY, JR, JG, and AO designed, performed, and analyzed the experiments. YY and AO wrote the manuscript.

## Conflict of Interest Statement

The authors declare that the research was conducted in the absence of any commercial or financial relationships that could be construed as a potential conflict of interest.
