# Supplementary material for: GlnR Activation Induces Peroxide Resistance in Mycobacterial Biofilms
Source: Front Microbiol. 2018 Jul 4;9:1428. doi: 10.3389/fmicb.2018.01428 (PMC6039565; doi:10.3389/fmicb.2018.01428)
Supplement: Supplementary file 1 [file Table_1.pdf]

Table S1: List of plasmids used in this study.

Name	Remarks	References
pJL37	P_{hsp60}-based expression vector, *kan*^{r}	(Ojha et al., 2005; Yang et al., 2017)
pMH94	L5-attp-based integrative vector, *kan*^{r}	(Ojha et al., 2005)
pYY90	pMH94 + *glnR* @ SacI & XbaI, *kan*^{r}	This study
pYY94	P*hsp60* -msmeg_0565-0572+ pMH94 @ XbaI	This study
pJV53-SacB	Plasmid containing inducible recombineering protein gp60 gp61 from phage Che9c and SacB cassette, kan^r	(Ojha et al., 2005)
pEM2	P*hsp60* -mCherry cloned in pMH94 @ XbaI, kan^r	(Yang et al., 2017)
pYL11	Hyg cassette+ pJL37 @ NheI& SpeI, Hyg^r	This study
pYY96	P*msmeg_2425* -Dendra2+ pYL11 @ XbaI & NheI	This study
pCre-SacB	Plasmid carrying Cre and SacB, *kan*^{r}	This study
