# Supplementary material for: GlnR Activation Induces Peroxide Resistance in Mycobacterial Biofilms
Source: Front Microbiol. 2018 Jul 4;9:1428. doi: 10.3389/fmicb.2018.01428 (PMC6039565; doi:10.3389/fmicb.2018.01428)
Supplement: Supplementary file 2 [file Table_2.pdf]

Table S2: List of mycobacterial strains used in this study.

Name	Remarks	References
mc^2155	High-Frequency Transformation strain of *M. smegmatis*	(Ojha et al., 2005)
ΔMSMEG_2425-2427	deletion of *MSMEG_2425-2427* in mc^2155, zeo^r,	(Yang et al., 2017)
ΔglnR	deletion of *MSMEG_5784* in mc^2155, zeo^r,	This study
ΔglnRcomp	*ΔglnR with pYY90*, zeo^r, kan^r	This study
Δgpr	deletion of *MSMEG_0565-0572* in mc^2155, zeo^r,	This study
Δgprcomp	*Δgpr with pYY94*, zeo^r, kan^r	This study
ΔsoxR	deletion of *MSMEG_5450* in mc^2155, zeo^r,	This study
ΔsoxRcomp	Recombineering *MSMEG_5450* in *ΔsoxR*, zeo^r,	This study
