# Supplementary material for: GlnR Activation Induces Peroxide Resistance in Mycobacterial Biofilms
Source: Front Microbiol. 2018 Jul 4;9:1428. doi: 10.3389/fmicb.2018.01428 (PMC6039565; doi:10.3389/fmicb.2018.01428)
Supplement: Supplementary file 3 [file Table_3.pdf]

Table S3: List of oligonucleotides used in this study.

Name	Sequence	Remarks
Smeg5784KoA	GCGTAGATGGCTGCTCCGAAATCGGT	For isogenic deletion of *MSMEG_glnR*
Smeg5784KoB	TGGTGAGGGAGATGAGGTCTGAAGAGCAGTAGATCCAACAAG	For isogenic deletion of *MSMEG_glnR*
Smeg5784KoC	GTTGAGGTGTGAGGTGTGCTGAAGCCAGTCAGTGACCTCCAC	For isogenic deletion of *MSMEG_glnR*
Smeg5784KoD	CGGATGCACGACGAGTTCGGCCAT	For isogenic deletion of *MSMEG_glnR*
Smeg0565-0572KoA	GTTCGCGGCGGTTTACCGCCTCTG	For isogenic deletion of *MSMEG_0565-0572*
Smeg0565-0572KoB	TGGTGAGGGAGATGAGGTCTGAAGGTGATGGATTCGTCGAAC	For isogenic deletion of *MSMEG_0565-0572*
Smeg0565-0572KoC	GTTGAGGTGTGAGGTGTGCTGAAGCACACTCCGCACACAGGA	For isogenic deletion of *MSMEG_0565-0572*
Smeg0565-0572KoD	AGGCCGAACAGAATCGTCATGACG	For isogenic deletion of *MSMEG_0565-0572*
Smeg5450KoA	TGAGCAGATCCGCCACCAGCAGCA	For isogenic deletion of *Msmeg_5450*
Smeg5450KoB	TGGTGAGGGAGATGAGGTCTGAAGTCAGTTCGTGCATGTCCA	For isogenic deletion of *Msmeg_5450*
Smeg5450KoC	GTTGAGGTGTGAGGTGTGCTGAAGCGCACGCCTCTGACACAT	For isogenic deletion of *Msmeg_5450*
Smeg5450KoD	GCGCGGGCTCATCGACCAGATTGT	For isogenic deletion of *Msmeg_5450*
Smeg5450KiA	TTCGAGTGTGCGCAGATGCGCCGA	For *Msmeg_5450comp*
Smeg5450KiB	TGGTGAGGGAGATGAGGTCTGAAGCACGCAACAACGACGCCC	For *Msmeg_5450comp*
Smeg5450KiC	GTTGAGGTGTGAGGTGTGCTGAAGGCGTCAGGCGTTTGAGTT	For *Msmeg_5450comp*
Smeg5450KiD	GAGCCTTGCACCGCCAGCTCCATG	For *Msmeg_5450comp*
glnRcompF	CCAGCAGGTCGTCTAGACCCATGTAGCGGCCGACAT	For plasmid pYY90
glnRcompR	CGTGAGCCCGGAATTCCACTCGGTGGAGGTCACT	For plasmid pYY90
Smeg_0572F	TCCCTGGAGAGACATATGCCGTTCGACGAATCC	For plasmid pYY94
Smeg_0572-65IR1	CGCACCAGGTACCAGCTGACCGACAGGCCG	For plasmid pYY94(PuvII)

Smeg_0572-65IF2	CGGCCTGTCGGTCAGCTGGTACCTGGTGCG	For plasmid pYY94(PuvII)
Smeg_0572-65IR2	GGCACCCGCGACGAGCTCATCGGGGTTCTC	For plasmid pYY94(SacI)
Smeg_0572-65IF3	GAGAACCCCGATGAGCTCGTCGCGGGTGCC	For plasmid pYY94(SacI)
Smeg_0572-65IR3	GGGATGGTCGACGAATTCGACGAGACGGAC	For plasmid pYY94(EcoRI)
Smeg_0572-65IF4	GTCCGTCTCGTCGAATTCGTCGACCATCCC	For plasmid pYY94(EcoRI)
Smeg_0565R	CCGTGATGGCTAGCAGCGATCGAGGCGAT	For plasmid pYY94
Smeg2425F	TCTTAGCCCTACCTGACGAA	For qPCR
Smeg2425R	AAAGTCCGCCGTAGAAGAAC	For qPCR
P2425F	CGTTCGAGCCTCTAGAGTTCGTCGACGCACTGCT	For plasmid pYY96
P2425R	AGGTAGGGCTAAGGATCCTTTGTGTGAACCTCCTTG	For plasmid pYY96
SmegSigA Forward	AAGGCGTCCGGCGACTTCGTGT	For qPCR
SmegSigA Reverse	TCCACCTCTTCTTCGGCGTTGAGC	For qPCR
Smeg0572F	TCCATCACCGAGAACATCAAG	For qPCR
Smeg0572R	AATAGCTTTCGCCGTCCTC	For qPCR
Smeg0571F	GACCTGGAACAGAATTTCGC	For qPCR
Smeg0571R	GATTGCCCAGTGACGAGAG	For qPCR
Smeg0570F	TGCCTGAGATGACCTTCG	For qPCR
Smeg0570R	GTCAGATGGTCGTGGATCAC	For qPCR
Smeg0569F	CTGGGACAACTTCACTCTCG	For qPCR
Smeg0569R	CTCGTCACGGGTCATGAAG	For qPCR
Smeg0568F	CGCTTCTATGACCTCACGAC	For qPCR
Smeg0568R	ATTCCTCGATGGTGCAGAAC	For qPCR
Smeg0567F	ACGTCAGCACACCATCAC	For qPCR
Smeg0567R	ATGAGAAACGGTGCGGTAG	For qPCR
Smeg0566F	AATCTGCGTTTCCTCGGG	For qPCR
Smeg0566R	TGATCTCGGCATGAACATCG	For qPCR
Smeg0565F	TGGCGTTGCTGACCTATTC	For qPCR
Smeg0565R	CGGGATTGATCTCACGGAAG	For qPCR
