# Supplementary material for: GlnR Activation Induces Peroxide Resistance in Mycobacterial Biofilms
Source: Front Microbiol. 2018 Jul 4;9:1428. doi: 10.3389/fmicb.2018.01428 (PMC6039565; doi:10.3389/fmicb.2018.01428)
Supplement: Supplementary file 5 [file Image_1.pdf]

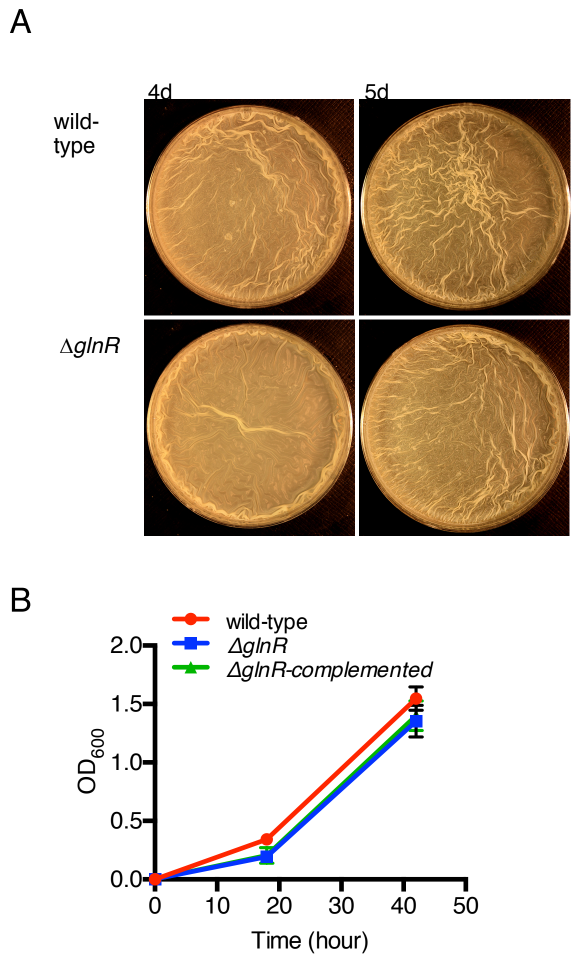

Figure S1: Growth of *M. smegmatis* in planktonic and biofilm cultures in modified M63 medium, referred to as biofilm medium. **A.** A top-down view of pellicle biofilms of wild-type and Δ*glnR* strains in detergent-free modified M63 medium at the indicated time point. **B.** Planktonic growth of wild-type , Δ*glnR* and Δ*glnR*-complemented strains in modified M63 medium with 0.05% (v/v) Tween80.
