# Supplementary material for: GlnR Activation Induces Peroxide Resistance in Mycobacterial Biofilms
Source: Front Microbiol. 2018 Jul 4;9:1428. doi: 10.3389/fmicb.2018.01428 (PMC6039565; doi:10.3389/fmicb.2018.01428)
Supplement: Supplementary file 6 [file Image_2.pdf]

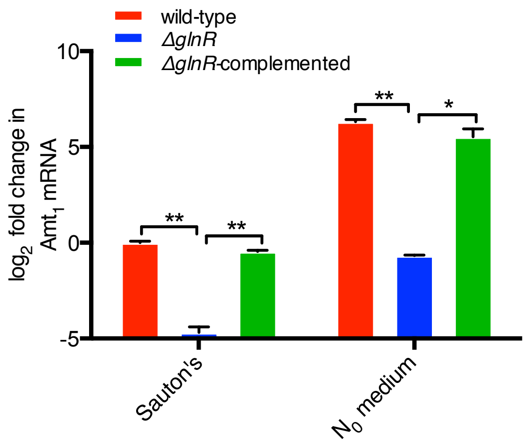

Figure S2: Activation of GlnR, measured by change in the transcript levels of Amt_1, after three hour exposure to nitrogen-free Sauton's medium (denoted as N_0 medium). A Δ*glnR* mutant and its complemented strains were used as controls. SigA transcript level was used as endogenous reference for normalization. Data represents mean ± SD of three biologically independent experiments.
