# Supplementary material for: GlnR Activation Induces Peroxide Resistance in Mycobacterial Biofilms
Source: Front Microbiol. 2018 Jul 4;9:1428. doi: 10.3389/fmicb.2018.01428 (PMC6039565; doi:10.3389/fmicb.2018.01428)
Supplement: Supplementary file 7 [file Image_3.pdf]

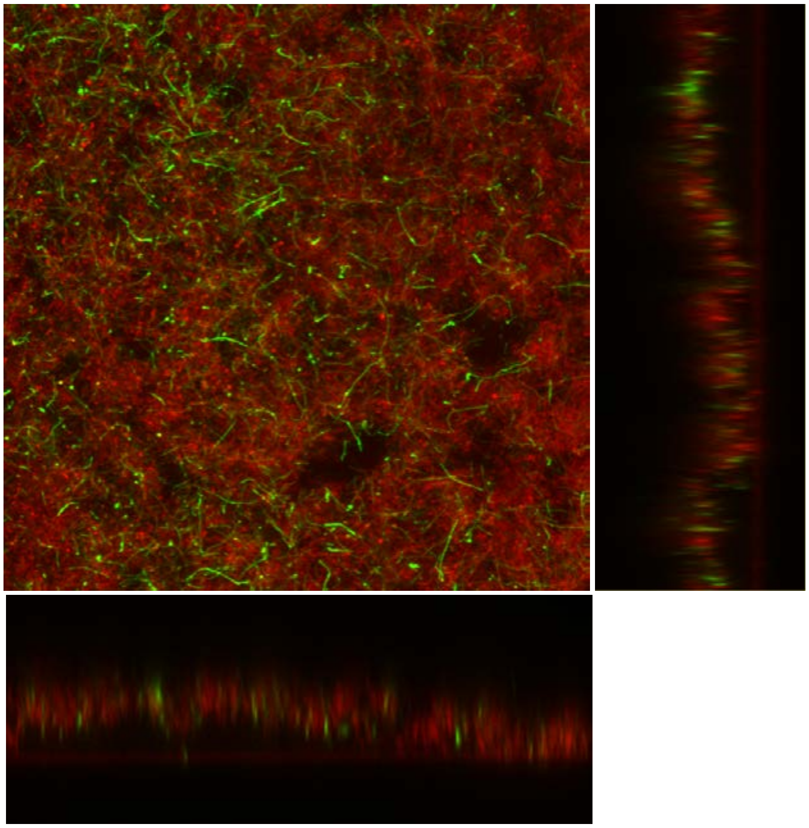

Figure S3: Expression of Dendra-2 (green) from the promoter of *amt*$_{1}$ (MSMEG_2425) in *M. smegmatis* biofilms after 4-days of growth in microfluidic device. *M. smegmatis* cells were constitutively expressing mCherry. The image in the mainframe shows maximum intensity projection of Dendra-2 (green) and mCherry (red) signals across z-stacks of biofilms. Orthogonal projections across the z-axis of two sides are shown in the insets.
