# Supplementary material for: GlnR Activation Induces Peroxide Resistance in Mycobacterial Biofilms
Source: Front Microbiol. 2018 Jul 4;9:1428. doi: 10.3389/fmicb.2018.01428 (PMC6039565; doi:10.3389/fmicb.2018.01428)
Supplement: Supplementary file 8 [file Image_4.pdf]

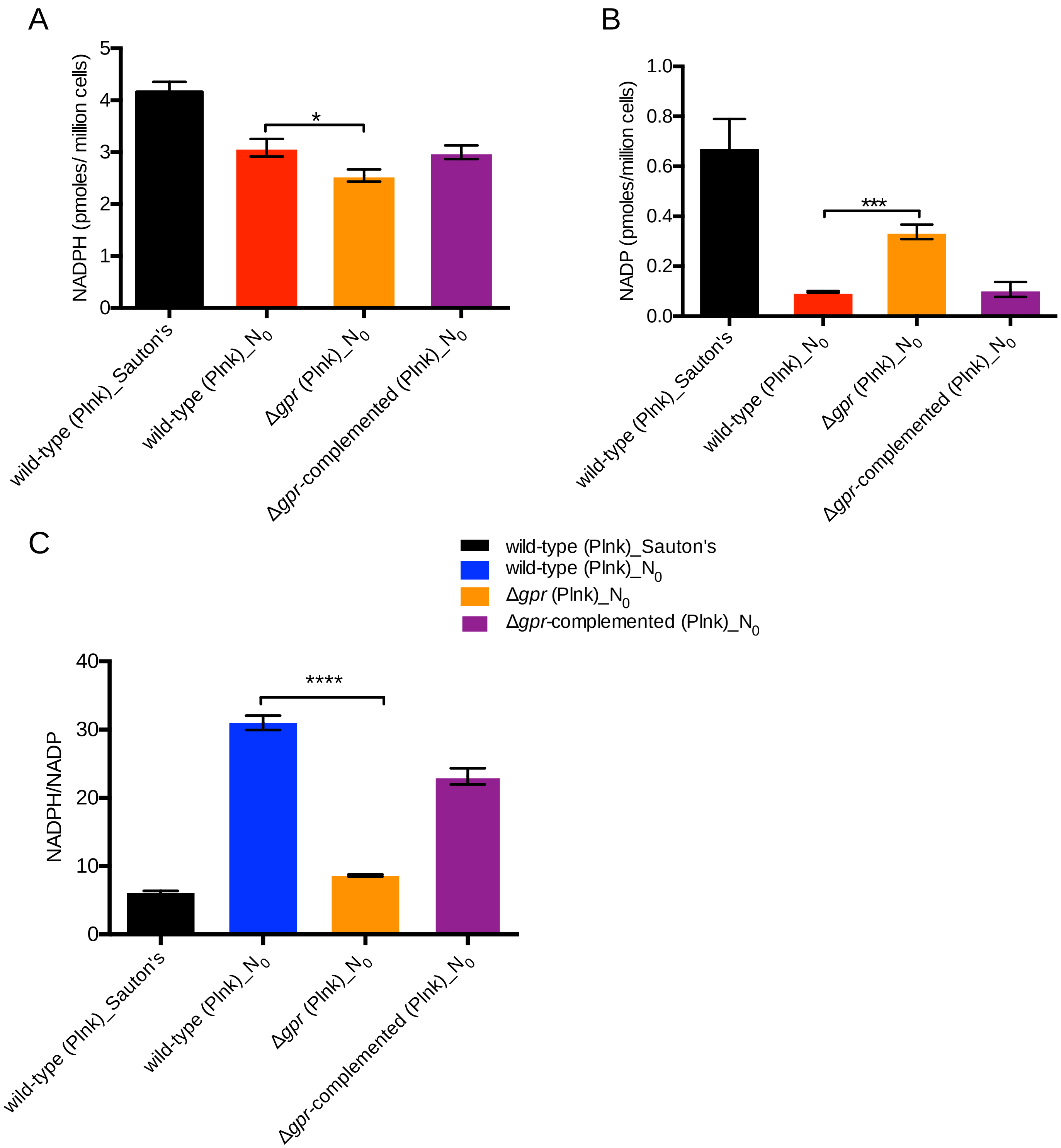

Figure S4: Levels of NADPH (A), NADP (B) and their ratio (C) in wild-type *M. smegmatis*, its isogenic *gpr* mutant, and its complemented derivative in nitrogen-free Sauton's medium. Value of wild-type in complete Sauton's medium was used as a reference. Data represent n = 3 ± SD. **** indicates $p < 0.0001$
