# Supplementary material for: GlnR Activation Induces Peroxide Resistance in Mycobacterial Biofilms
Source: Front Microbiol. 2018 Jul 4;9:1428. doi: 10.3389/fmicb.2018.01428 (PMC6039565; doi:10.3389/fmicb.2018.01428)
Supplement: Supplementary file 9 [file Image_5.pdf]

A

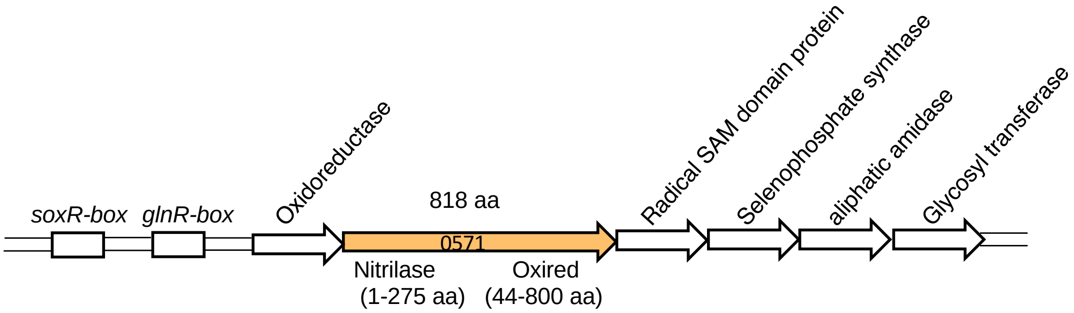

B

M T T L A A V S A N F T R D L E Q N F A L I A D L A E Q A R E K G V D F L V L P E A A I G G Y L S S L G
N H G D T V R A T S R S L P P A I R L D G P E I A R V Q Q I V G D L V V A I G F C E L A D D G E T R Y N
A A A V L D R F D V Y G S Y R K V H Q P L G E G M S Y S A G S D Y G V F D T P V G R V G L Q I C Y D K
A F P E A A R L M A L G G A Q I I A S L S A W P A A R T A T A Q N L Q D D R W T Y R F N L F D M A R A
L D N Q V F W V A S N Q S G S F G S L R Y V G N A K V V D P G G N V L A T T L L D S G M A V A E V D I
D E T F R T M R A G M F H L R D R R P D V Y A P L T D S D G P R T A K W R E L A H A U D D L R S P V
A G R Q H P A L L F P E P G D P R P S D R G N A L R R R R F R G A L E P S T G R G E R A G T R Q V R
L R L H L G G R D P S A H R Q R R P A V Q R G C G A R G R R H A P R T G A V M T T H V P V A V I G G
G Q A G L S V S W Y L V R A G I E H I V I E S K T P M H A W A D T R W D N F T L V T P N W H C R L P G
Y P Y A G P D P D G F M T R D E V V D W L A G W L D T F D P P L R N H T Q V T R L Q N R A G G G F E
V T L R D E S G Q S T L T C D H A V I A T G G Y P V P V I P S Y A A Q L D E T I L Q I H S E Q Y R N A G T
L P D G A V L V V G T G Q S G A Q I A E D L H L A G R R V H L A V G G A P R V A R F Y R G R D C M T
W L A D M G V Y D R P A Q Q Y P G G Q A A I E K T N H Y V T G R D G G R D V D L R Q F A T E G M R L
Y G T L A D G K D S T L R F E P T L A E A L D H A D S V Y N S I C S D I D A H I E R N G I D A P P A S R Y
E P V W K P E T E T T T L D L A A A G I T S I V W A I G Y R P D Y R W I A A S A F D G A G R P M Q T R G
I T N V A G L S F V G L P W M H T W G S G R F L G I D R D A R H I A A T I I S S Y H E S V L R L A M K V

Figure S5: Possibility of MSMEG_0571 in *gpr* locus as a new selenoprotein. **A.** Schematic representation of new ORFs due to incorporation of selenocysteine at the UGA codon of MSMEG_0571, resulting in a single polypeptide of 818 amino acids, terminating at the UAG codon of original MSMEG_0569 ORF. BLAST search of this protein identifies a nitrilase and flavin-dependent oxidoreductase (Oxired) domains at the N- and C- terminals, respectively. The position of amino acids encompassing the domains are also indicated. **B.** Sequence of the possible selenoprotein encoded by *gpr*. Seleneocysteine (U) is indicated in red, while the segments of the protein that were originally parts of MSMEG_0571 (blue) and MSMEG_0569 (brown) are color coded. A linker (black) between the two is out of frame with the originally annotated MSMEG_0570.
